# Case Report: Complete remission of Guillain-Barré syndrome in neuropsychiatric lupus with telitacicept

**DOI:** 10.3389/fimmu.2026.1726901

**Published:** 2026-01-23

**Authors:** Yunqi Bao, Wei Zhang, Xia Liu, Nannan Gai

**Affiliations:** Department of Rheumatology, Xi’an Fifth Hospital, Xi’an, Shaanxi, China

**Keywords:** B-cell targeted therapy, biological therapy, dual BLyS/APRIL inhibition, Guillain-Barré syndrome, neuropsychiatric systemic lupus erythematosus, telitacicept

## Abstract

Neuropsychiatric systemic lupus erythematosus (NPSLE) manifesting as Guillain-Barré syndrome (GBS) is exceptionally rare, with only 28 documented cases in medical literature, and current biological therapies show limited efficacy. We report a 34-year-old woman who presented with progressive quadriparesis, bilateral ptosis, and facial palsy developing over one month. Laboratory investigations revealed marked B-cell dysregulation with elevated immunoglobulin G (24.30 g/L, normal 7-16), positive antinuclear antibodies (1:320), anti-double-stranded DNA antibodies (128 IU/ml, normal <20), anti-ribosomal P antibodies (450 AU/ml, normal <20), and hypocomplementemia (C3 1.10 g/L, C4 0.28 g/L). Cerebrospinal fluid analysis showed cytoalbuminologic dissociation (protein 1314 mg/L, cells 1×10^6^/L), and nerve conduction studies confirmed acute inflammatory demyelinating polyradiculoneuropathy. Following inadequate response to intravenous immunoglobulin monotherapy, combination treatment was initiated with methylprednisolone (40mg daily), cyclophosphamide (0.6g biweekly), hydroxychloroquine, and telitacicept (160mg weekly), a novel dual inhibitor of B lymphocyte stimulator and a proliferation-inducing ligand that simultaneously targets B cells and plasma cells. At six-month follow-up, the patient achieved complete neurological recovery with significant laboratory improvements: immunoglobulin G decreased 61.6% to 9.34 g/L, anti-double-stranded DNA antibodies decreased 82.8% to 22 IU/ml, erythrocyte sedimentation rate normalized from 63 to 8 mm/h, and complement levels recovered. Corticosteroids were successfully tapered to 4mg daily without disease flare. This first report of telitacicept use in NPSLE-GBS demonstrates that dual BLyS/APRIL inhibition can achieve complete remission in refractory cases, offering a promising therapeutic approach that warrants further investigation in controlled trials.

## Introduction

Neuropsychiatric involvement affects 12-95% of lupus patients (1). The American College of Rheumatology recognizes 19 distinct neuropsychiatric syndromes. Among these, acute inflammatory demyelinating polyradiculoneuropathy mimicking Guillain-Barré syndrome is exceptionally rare. A systematic review identified only 28 documented cases of systemic lupus erythematosus (SLE)-related GBS, with 57.1% presenting before SLE diagnosis (2). This emphasizes the importance of autoimmune screening in atypical GBS presentations.

Unlike idiopathic GBS, SLE-related GBS demonstrates higher cranial nerve involvement (up to 60%), more axonal variants, and poorer response to standard therapy (3, 4). Treatment outcomes differ substantially: IVIG achieves only 42.9% response in SLE-GBS versus 80-90% in idiopathic GBS, while cyclophosphamide shows 85.7% efficacy (2). These observations indicate SLE-GBS requires strategies addressing both demyelination and systemic autoimmunity.

The pathogenesis of NPSLE is complex. It involves autoantibodies, complement activation, and inflammatory cytokines (5). In GBS-like cases, antibodies likely attack peripheral nerves directly (6). Standard treatment with intravenous immunoglobulin often shows variable responses in SLE-related GBS (2).

B lymphocyte stimulator (BLyS/BAFF) and a proliferation-inducing ligand (APRIL) regulate B-cell survival and antibody production (7). Both are overexpressed in SLE patients (8). Telitacicept is a TACI-Fc fusion protein that inhibits both BLyS and APRIL simultaneously, representing the first dual-target biological agent (9). It received approval in China for SLE treatment in 2021. Full approval followed in November 2023 after successful Phase III trials (9, 10). The drug showed impressive results with 82.6% of patients achieving SRI-4 response versus 38.1% on placebo (11). While effective in other neurological autoimmune conditions (12, 13), its use in NPSLE has not been previously reported.

Here, we report the first case of NPSLE presenting as GBS treated with telitacicept-containing therapy, aiming to describe clinical efficacy and safety in this rare but severe lupus manifestation.

## Case description

### Initial presentation

A 34-year-old Chinese woman presented in November 2024 with one-month progressive neurological symptoms. Initial manifestations included bilateral fingertip numbness extending to palms and buttocks, accompanied by taste disturbance. She developed proximal muscle weakness (Medical Research Council [MRC] grade 3 proximally, 4+ distally in lower limbs; 4+ in upper limbs), bilateral ptosis, facial asymmetry, and mild dysphagia. She denied rash, photosensitivity, oral ulcers, or Raynaud phenomenon. Medical and family histories were unremarkable.

Neurological examination confirmed the muscle weakness pattern and revealed diminished deep tendon reflexes throughout. Cranial nerve examination revealed: bilateral partial ptosis (cranial nerve III involvement) with intact pupillary responses and full extraocular movements without diplopia; bilateral facial weakness (cranial nerve VII) with incomplete eye closure and asymmetric smile; and diminished gag reflex with mild dysphagia (cranial nerves IX/X). Cranial nerves IV and VI were intact, and no ophthalmoplegia was observed.

Cognitive assessment revealed preserved higher mental functions with a Mini-Mental State Examination (MMSE) score of 29/30. The patient was alert, fully oriented, and demonstrated no evidence of psychosis, mood disturbance, or cognitive impairment.

Initial laboratory investigations at an outside hospital revealed leukopenia (white blood cell count 2.83×10^9^/L), elevated erythrocyte sedimentation rate (45 mm/h), positive antinuclear antibodies (ANA), and markedly elevated immunoglobulin G (IgG 23.2 g/L, normal 7–16 g/L).

### Diagnostic workup

Brain magnetic resonance imaging (MRI) was normal. Nerve conduction studies demonstrated abnormal F-waves, prolonged H-reflexes, absent blink reflexes, and bilateral facial nerve involvement, consistent with acute inflammatory demyelinating polyradiculoneuropathy. Cerebrospinal fluid (CSF) analysis revealed cytoalbuminologic dissociation (protein 1314 mg/L, cells 1×10^6^/L).

Comprehensive infectious workup was negative, including human immunodeficiency virus (HIV) antibody, hepatitis B surface antigen (HBsAg), anti-hepatitis C virus (HCV) antibody, anti-Epstein-Barr virus (EBV) immunoglobulin M (IgM), and anti-cytomegalovirus (CMV) IgM. CSF viral panel (herpes simplex virus polymerase chain reaction [PCR], varicella-zoster virus PCR, enterovirus PCR) was also negative, excluding infectious etiologies.

Metabolic screening excluded diabetic neuropathy (fasting glucose 4.8 mmol/L, HbA1c 5.2%).

The patient received intravenous immunoglobulin (IVIG) 25g daily for 5 days at another institution with partial improvement. Given incomplete response and systemic features, she was referred to our rheumatology department.

### Diagnosis and treatment initiation

Comprehensive immunological assessment at our institution revealed: ANA 1:320 (cytoplasmic pattern), anti-double-stranded DNA antibodies 128 IU/ml (normal <20), anti-ribosomal P protein antibodies 450 AU/ml (normal <20), complement C3 1.10 g/L (normal 1.15-1.50), complement C4 0.28 g/L (normal 0.35-0.45), and IgG 24.30 g/L. Complete blood count showed persistent leukopenia (2.9×10^9^/L) with lymphopenia (0.65×10^9^/L). ESR was 63 mm/h and C-reactive protein 2.35 mg/L. The patient fulfilled the Systemic Lupus International Collaborating Clinics (SLICC) classification criteria for SLE based on neurological involvement, leukopenia, positive ANA, and anti-dsDNA antibodies.

Following the diagnosis of neuropsychiatric SLE presenting as GBS, treatment was initiated with methylprednisolone 40mg daily, cyclophosphamide 0.6g biweekly, and hydroxychloroquine 200mg twice daily. Given the marked B-cell hyperactivity evidenced by hypergammaglobulinemia and high-titer autoantibodies, telitacicept 160mg weekly subcutaneously was added after obtaining informed consent and discussing the limited published experience in neuropsychiatric lupus.

### Clinical outcomes

After six cyclophosphamide cycles (total 3.6g), muscle strength normalized (grade 5/5) by May 2025. Ptosis and facial weakness resolved completely. Laboratory improvements included: ESR 63→8 mm/h, IgG 24.30→9.34 g/L (61.6% reduction), anti-dsDNA 128→22 IU/ml (82.8% reduction), C3 normalized to 1.28 g/L, WBC normalized to 5.5×10^9^/L.

Neurological recovery followed a progressive pattern: by week 2 (after 2nd cyclophosphamide dose), proximal lower limb strength improved from grade 3 to 4/5 and ptosis began resolving; by week 8, facial weakness resolved completely and dysphagia improved significantly; by month 4, limb strength normalized to grade 5/5 in all extremities; by month 6, sensory symptoms (fingertip numbness and dysgeusia) resolved completely. Deep tendon reflexes, initially diminished throughout, returned to normal by month 5.

Methylprednisolone was tapered from 40mg to 14mg post-cyclophosphamide, then to 4mg daily by May 2025. Maintenance therapy included mycophenolate mofetil 0.5g twice daily, hydroxychloroquine, and continued telitacicept. The timeline of clinical events and treatment response is summarized in **Table 1**.

**Table 1:** Timeline of clinical events and treatment response.

Date	Clinical event	Laboratory findings	Treatment
Oct 2024	Onset: fingertip numbness, taste disturbance	–	Self-medication (tianma tablets)
Nov 2024 (early)	Progressive weakness, ptosis, facial palsy	WBC 2.83×10^9^/L, ESR 45 mm/h	–
Nov 15-19, 2024	Partial improvement	CSF protein 1314 mg/L, cell count 1×10^6^/L	IVIG 25g × 5 days
Nov 25, 2024	Referred to our institution	ANA 1:320 (cytoplasmic pattern), anti-double-stranded DNA antibodies 128 IU/ml, anti-ribosomal P protein antibodies 450 AU/ml, IgG 24.30 g/L, C3↓, C4↓	–
Nov 28, 2024	Treatment initiation	ESR 63 mm/h	MP 40mg, CTX, HCQ, telitacicept
Dec 12, 2024	Improved strength	ESR 14 mm/h	2nd CTX dose
Feb 10, 2025	Near-normal strength	ESR 8 mm/h	MP tapered to 16mg
May 13, 2025	Complete recovery	IgG 9.34 g/L, ESR 8 mm/h, anti-dsDNA 22 IU/ml, C3 1.28 g/L	MP 4mg, MMF maintenance

MP, methylprednisolone; CTX, cyclophosphamide; HCQ, hydroxychloroquine; MMF, mycophenolate mofetil.

Throughout the treatment period, complete blood counts were monitored biweekly, and liver and kidney functions were assessed monthly.

Treatment was well-tolerated. Adverse events included one mild upper respiratory infection (week 6, resolved spontaneously) and transient injection site erythema (week 2, <24 hours). No serious infections, hepatotoxicity, or nephrotoxicity occurred despite IgG reduction to 9.34 g/L.

## Discussion

This represents the first reported use of telitacicept in NPSLE manifesting as GBS. According to the systematic review by Xiong et al., 57.1% of SLE-related GBS cases present before SLE diagnosis, highlighting the importance of screening for systemic autoimmune disease in atypical GBS presentations (2). The inadequate IVIG response observed is consistent with previous reports (2, 14).

Anti-ribosomal P antibodies are found in 90% of NPSLE cases and correlate with disease severity (15). The combination of these antibodies with markedly elevated IgG levels (24.30 g/L) indicated severe B-cell dysregulation, justifying aggressive B-cell targeted therapy.

### Treatment response analysis

Among 28 reported SLE-GBS cases, cyclophosphamide showed 85.7% efficacy while IVIG monotherapy succeeded in only 42.9% (2). Our patient’s pattern—poor IVIG response followed by improvement with combination therapy—aligns with these findings. The addition of telitacicept represents a novel approach.

The choice of moderate-dose methylprednisolone (40mg daily) rather than pulse therapy (500-1000mg daily for 3–5 days) warrants discussion. While pulse steroids have demonstrated efficacy in SLE-related GBS and are recommended by some authorities, we opted for moderate dosing given the patient’s baseline leukopenia (2.9×10^9^/L) and lymphopenia (0.65×10^9^/L), which increased infection risk. The concurrent initiation of cyclophosphamide provided rapid immunosuppression. This approach achieved complete remission, though pulse steroids remain a valid alternative, particularly in patients without cytopenia.

Plasmapheresis was not attempted following partial IVIG response. While plasmapheresis is equally effective to IVIG in idiopathic GBS, several factors influenced this decision: the partial response to IVIG suggested some therapeutic effect; recognition of underlying SLE necessitated treatment targeting the autoimmune etiology rather than symptomatic GBS management alone; plasmapheresis provides transient antibody removal without addressing ongoing autoantibody production; and the established efficacy of cyclophosphamide in SLE-related GBS (85.7% response rate) supported this approach.

The 61.6% reduction in IgG levels observed in our patient exceeds typical responses to conventional immunosuppression. In the Phase III trial of telitacicept for SLE, mean IgG reduction was approximately 30% at week 52 (10). This enhanced response may reflect synergy with cyclophosphamide or increased efficacy in patients with baseline hypergammaglobulinemia. The 82.8% reduction in anti-dsDNA antibodies further supports robust immunological response.

### Mechanistic insights

The pathogenesis of NPSLE involves both inflammatory and vascular mechanisms, with autoantibodies, immune complexes, and complement activation playing crucial roles (5). In GBS-like presentations, molecular mimicry likely drives nerve damage (16). Telitacicept’s dual BLyS/APRIL inhibition offers theoretical advantages: comprehensive targeting of both B cells and plasma cells (unlike rituximab which spares CD20-negative plasma cells) (17), prevention of compensatory pathway upregulation (18), and potential for sustained disease modification evidenced by complement normalization despite minimal steroid dosing.

The prolonged continuation of telitacicept warrants clarification. Although not specifically indicated for NPSLE, telitacicept is approved for SLE treatment in China. Continued therapy was justified by: (1) the need for sustained B-cell suppression given baseline hypergammaglobulinemia and high-titer autoantibodies suggesting high relapse risk; (2) successful steroid tapering to 4mg daily without disease flare, achieving a major therapeutic goal; (3) its potential role in preventing neurological relapse through ongoing autoantibody suppression. The observed 82.8% reduction in anti-dsDNA antibodies suggests sustained immunomodulation. However, optimal duration of telitacicept in NPSLE remains undefined and requires prospective study.

Recent studies support telitacicept’s efficacy in neurological autoimmune conditions. It demonstrated effectiveness in rituximab-refractory neuromyelitis optica (19) and achieved 98.1% response rates in myasthenia gravis trials (13). These findings suggest dual BLyS/APRIL inhibition may be particularly effective for antibody-mediated neurological disorders.

The safety profile aligns with Phase III data showing infection rates of 28.3% (vs 25.4% placebo) and injection site reactions in 5.7% (11). Monthly immunoglobulin monitoring is recommended, with IVIG supplementation considered if IgG falls below 4 g/L.

### Study limitations

Several limitations must be acknowledged. As a single case report, causality cannot be definitively established.

Critically, the concurrent use of cyclophosphamide and telitacicept precludes attribution of therapeutic benefit to either agent alone, and individual drug efficacy cannot be established from this case. The combination was employed because cyclophosphamide has demonstrated 85.7% efficacy in SLE-GBS, and withholding this proven therapy to evaluate a novel agent would have been ethically unjustifiable given the severity of neurological involvement. Future controlled studies comparing telitacicept monotherapy or combination regimens are essential to determine its independent contribution.

The relatively short follow-up period limits assessment of long-term durability and potential late adverse effects. Optimal dosing and treatment duration for telitacicept in NPSLE remain undefined. Additionally, lack of historical controls or matched comparisons limits efficacy assessment.

### Future directions

This case suggests several research priorities. Prospective studies should evaluate telitacicept in NPSLE, particularly comparing efficacy with rituximab and belimumab. Biomarker studies could identify patients most likely to benefit from dual BLyS/APRIL inhibition. Investigation of telitacicept monotherapy versus combination therapy would clarify its independent contribution. Long-term safety data in NPSLE patients is essential.

The successful steroid tapering to 4mg daily addresses a major therapeutic goal in SLE management, potentially reducing steroid-related morbidity. Early implementation of B-cell targeted therapy should be considered in patients with markers of B-cell dysregulation.

## Conclusions

This case demonstrates favorable outcomes in NPSLE-GBS treated with a telitacicept-containing regimen. Complete neurological recovery, substantial autoantibody reduction, and successful steroid minimization were achieved. While causality cannot be definitively established from a single case, these findings support further investigation of telitacicept for refractory NPSLE, particularly in patients with prominent B-cell involvement. Systematic studies are warranted to establish optimal treatment protocols for this challenging manifestation of SLE.

## Patient perspective

The patient expressed satisfaction with treatment outcomes. She emphasized relief at avoiding long-term high-dose corticosteroids and appreciated rapid functional recovery enabling work resumption. Weekly injections were well-tolerated without injection site reactions.

## Data Availability

The original contributions presented in the study are included in the article/supplementary material. Further inquiries can be directed to the corresponding author.
